# Supplementary material for: Vascular invasion-associated gene expression is detectable in pre-surgical biopsies of stage I lung adenocarcinoma
Source: Nat Commun. 2026 Mar 24;17:2581. doi: 10.1038/s41467-026-70600-2 (PMC13013712; doi:10.1038/s41467-026-70600-2)
Supplement: Supplementary file 1 — Supplementary Information [file 41467_2026_70600_MOESM1_ESM.pdf]

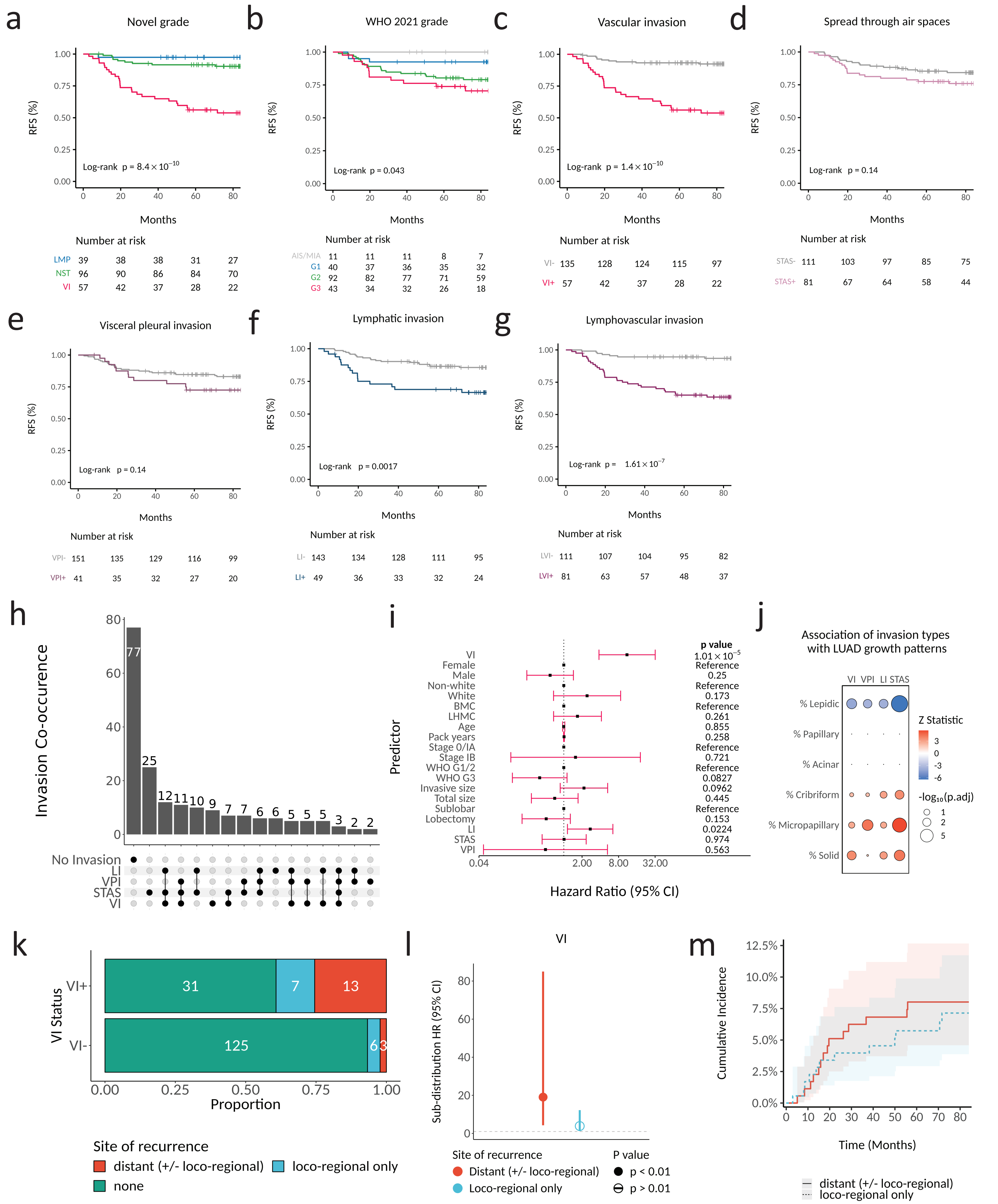

Supplementary Fig. 1. VI is the stage I LUAD invasion type most associated with recurrence.

Supplementary Fig. 1. VI is the stage I LUAD invasion type most associated with recurrence. a. Association of novel grading (n=192 tumors) and **b**. WHO grading (n=186 tumors) systems with 7-year RFS in the stage I LUAD discovery cohort. Mucinous tumors (n=6) were excluded from WHO grading. LMP, low malignant potential; NST, no special type; VI, vascular invasion. Survival curves represent Kaplan-Meier estimates of RFS. P values were calculated by two-sided log-rank tests comparing survival distributions across groups. **c-g**. Association of VI, STAS, VPI, LI, and LVI pathology, respectively, with 7-year RFS in the stage I LUAD discovery cohort (n=192 tumors). STAS, spread through air spaces; VPI, visceral pleural invasion; LI, lymphatic invasion; LVI lymphovascular invasion. Survival curves represent Kaplan-Meier estimates of RFS. P values were calculated using two-sided log-rank tests comparing survival distributions between groups. **h**. Co-occurrence of VI, STAS, VPI, and LI in the stage I LUAD discovery cohort (n=192 tumors). **i**. Association of VI with 7-year RFS after adjustment for clinical covariates, collection site (LHMC, Lahey Hospital & Medical Center; BMC, Boston Medical Center) and other invasion types. Hazard ratios (HRs) and 95% confidence intervals (CI) were estimated using a multivariable Cox proportional hazards regression model (n=176 tumors, n=31 events). Covariates included gender, race, site, age, smoking pack-years, pathologic stage (TNM 8th edition), WHO grade, invasive and total tumor size, surgical procedure, LI, STAS, and VPI. Patients with any missing covariates (n=10) were excluded: including n=3 excluded for missing race, and n=7 for missing pack years. Additionally, patients with mucinous tumors (n=6) were excluded. P values are two-sided Wald test P values derived from the Cox model. A sensitivity analysis using a reduced covariate set including all tumors (n=192) yielded similar results. **j**. Association between invasion types and LUAD growth pattern in the stage I LUAD discovery cohort (n=192 tumors). For each invasion type, the percentage of each growth pattern was compared between invasion negative and invasion positive tumors using two-sided Wilcoxon rank-sum tests. P values were Holm-Bonferroni adjusted across all invasion-pattern comparisons. Dot size represents $-\log_{10}$(adjusted P value), and dot color indicates the signed Wilcoxon Z statistic (red, higher in invasion-positive tumors; blue, higher in invasion-negative tumors). **k**. Recurrence type in VI^- and VI^+ cases (n=185 tumors). Numbers within bars indicate absolute case counts. Patients with unknown recurrence location (n=7) were excluded. **l**. Recurrence site-specific sub-distribution hazard ratio (HR) for VI in the stage I LUAD discovery cohort (n=185 tumors). Patients with unknown

recurrence site (n=7) were excluded. Sub-distribution HRs were estimated using multivariable Fine-Gray regression. Models were adjusted for gender, age, pack years, surgical procedure, and collection site. Points indicate sub-distribution HRs and vertical lines designate the 95% CI. Two-sided Wald tests were used to assess statistical significance. Exact p values were P = 1.1×10^{-4} for distant (± locoregional) recurrence and P = 0.022 for loco-regional-only recurrence. **m.** Cumulative incidence function for recurrence type (n=185 tumors). Shaded regions indicate pointwise 95% CIs. Substantial overlap of CIs indicates no clear separation between recurrence sites over time. Source data are provided as a Source Data file.

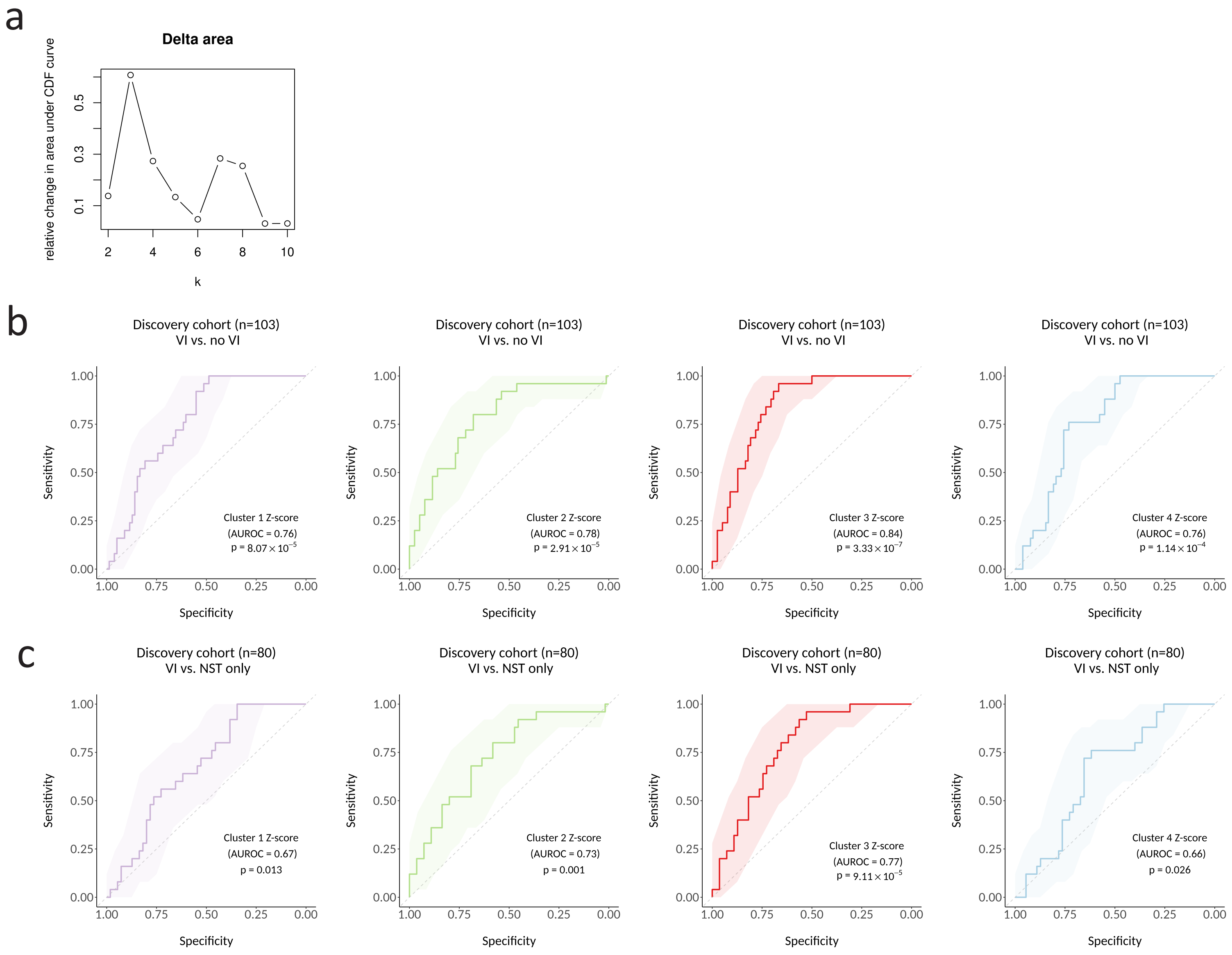

Supplementary Fig. 2. The four VI gene expression clusters individually predict VI in the discovery cohort even in the absence of LMP tumors.

Supplementary Fig. 2. The four VI gene expression clusters individually predict VI in the discovery cohort even in the absence of LMP tumors. a. Change in area under the cumulative density function (CDF) when varying the number of clusters (k=2-10) for consensus clustering of the VI gene expression clusters. K=4 was selected for a large change in delta under the CDF up to but not beyond k=4. **b**. ROC curves showing per sample cluster score discrimination between VI^+ and VI^- tumors in the discovery cohort (n=103 tumors). Shaded regions denote the pointwise 95% CIs of sensitivity at fixed specificity values, computed by stratified bootstrap resampling (2,000 replicates). Statistical significance was assessed using a two-sided Wilcoxon rank-sum test comparing per-sample cluster scores between VI^+ and VI^- tumors. **c**. ROC curves showing per sample cluster score discrimination between VI^+ and NST tumors in the discovery cohort (n=80 tumors), excluding LMP tumors. CIs and statistical testing were performed as in (b). Source data are provided as a Source Data file.

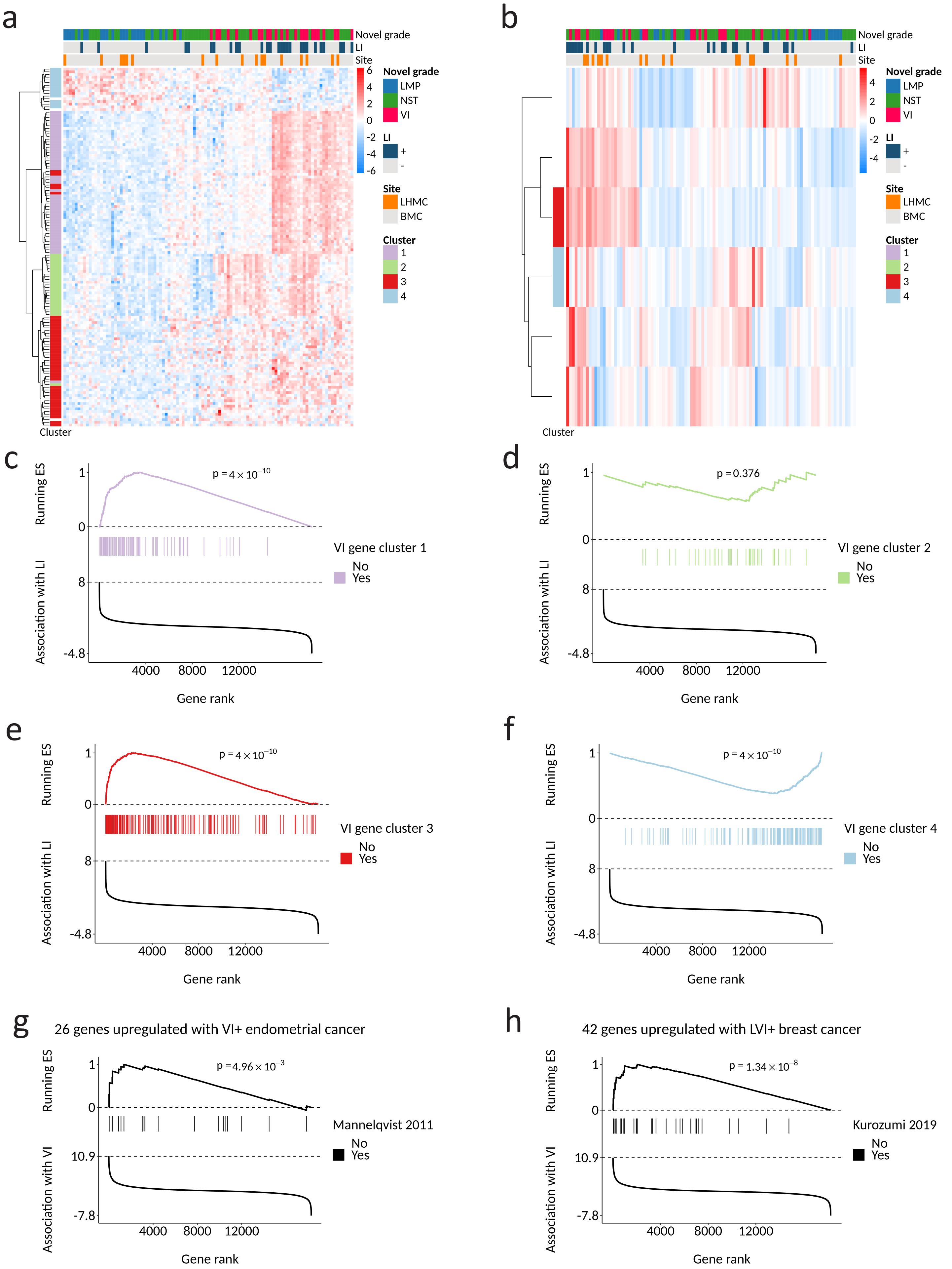

Supplementary Fig. 3. VI but not LI is associated with expression of tissue remodeling genes.

Supplementary Fig. 3. VI but not LI is associated with expression of tissue remodeling genes. a. Co-expression heatmap of 133 genes differentially expressed between VI and LMP (FDR < 0.01) in the discovery cohort of stage I LUAD (n=103 tumors) when including LI as a covariate. Genes are annotated by the VI gene expression clusters defined in Fig. 1b. Genes labeled in white are not present in the Fig. 1b. heatmap. Heatmap units are log counts per million (CPM) scaled by transcript. **b**. Heatmap of 6 genes associated with LI (FDR < 0.01). Genes are annotated if they belong to one of the clusters defined in Fig. 1b. **c-f**. Gene-set enrichment analysis (GSEA) of the four VI gene clusters from Fig. 1b. within all genes ranked by strength and direction of association with LI. P values were derived from permutation-based GSEA and adjusted using Bonferroni correction across the four tested gene sets. **g.** GSEA of 26 genes previously reported to be upregulated in VI^+ endometrial cancer (Mannelqvist et al., 2011) against a ranked list of all genes ranked by strength and direction of association with VI. **h.** GSEA of 42 genes found to be upregulated in LVI^+ breast cancer (Kurozumi et al., 2019) against a ranked list of genes ordered by association with VI. For panels g-h, gene-set-level p values were calculated by GSEA and Bonferroni-adjusted. Source data are provided as a Source Data file.

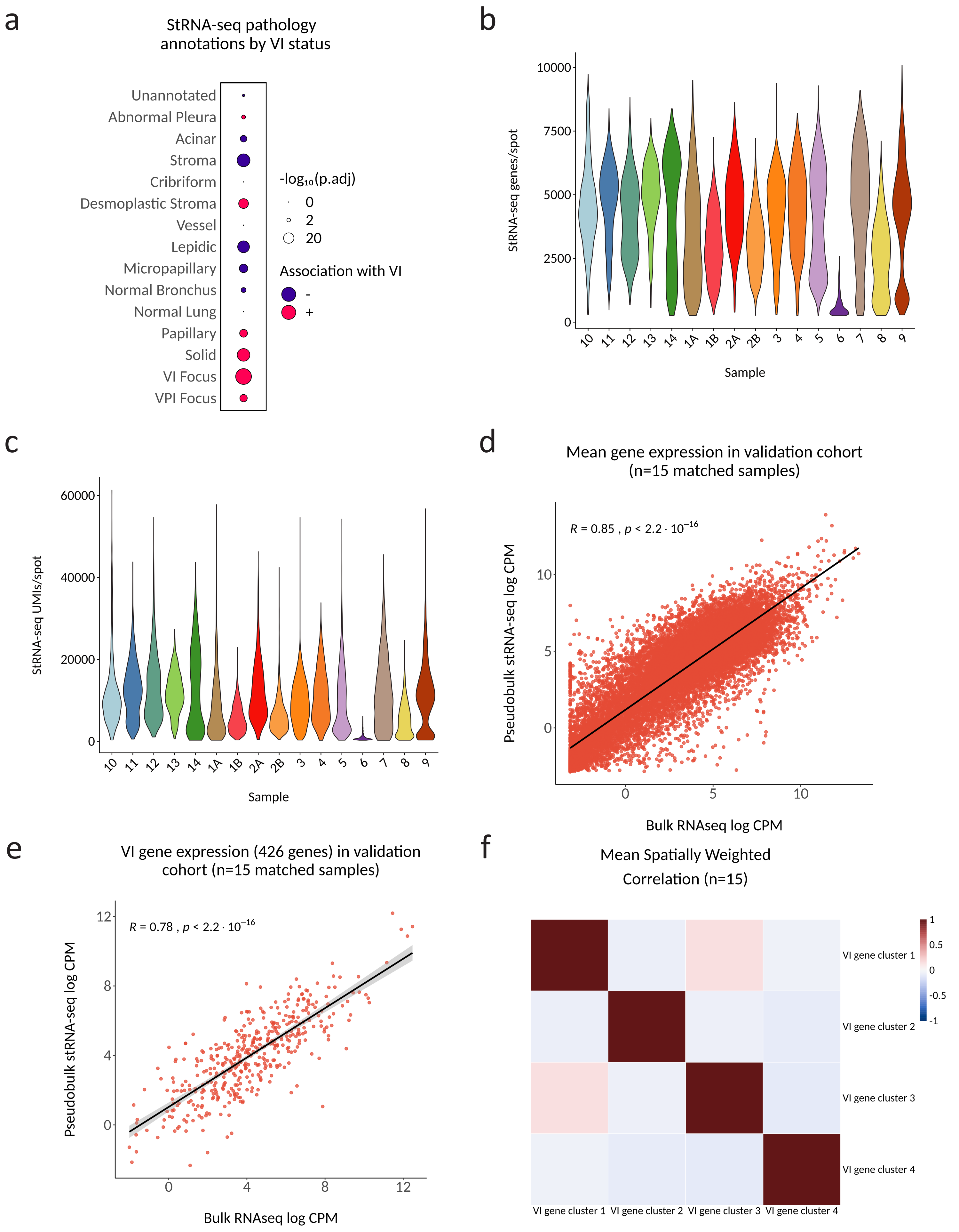

Supplementary Fig. 4. StRNA-seq quality control and concordance with bulk RNA-seq.

Supplementary Fig. 4. StRNA-seq quality control and concordance with bulk RNA-seq. a. Association of spot-wise pathology annotations with tumor-level VI status in stRNA-seq samples that passed quality control (QC) (n=15 samples). For each pathology annotation type, spots were down sampled to equal counts per annotation and aggregated at the sample level. Enrichment or depletion of each pathology annotation type in VI^{+} (n=7 samples) vs. VI^{-} (n=8 samples) was assessed using chi-square test. P values were adjusted for multiple testing across pathology categories using the Holm-Bonferroni adjustment and are displayed as $-\log_{10}$(p.adj). Red indicates over-representation and blue indicates under-representation in VI^{+} tumors. **b**. Number of detected genes per spot and **c**. UMI counts per spot by sample in the stRNA-seq data prior to QC (n=16). Violin plots show the distribution of features per spot for each sample. Sample 6 failed QC and was excluded from downstream analysis. **d**. Correlation of mean gene expression between pseudo-bulked stRNA-seq data and bulk RNA-seq data (n=15 matched samples). Each point represents a gene detected in both stRNAseq and bulk RNA-seq, with values corresponding to mean log-CPM expression across samples. The solid line indicates the linear regression fit, with the shaded region showing the 95% CI. Spearman rank correlation coefficient and the associated two-sided P value are shown. **e.** Correlation of VI gene signature expression (426 genes) shared between pseudo-bulked stRNA-seq data and bulk RNA-seq data (n=15 matched samples). Each point represents one VI signature gene, with values corresponding to mean log-CPM expression across samples. The solid line indicates the linear regression fit, with the shaded region showing the 95% CI. Spearman rank correlation coefficient and the associated two-sided P value are shown. **f**. Mean spatially weighted correlation of spot-wise VI gene cluster enrichment scores across all stRNA-seq samples (n=15 samples). For each sample, spatially weighted Spearman correlation coefficients were computed between VI gene cluster scores at each spot using geographically weighted summary statistics with a bandwidth=5, accounting for spatial proximity between spots. Heatmap values indicate the mean spatially weighted Spearman correlation coefficients averaged across samples. Source data are provided as a Source Data file.

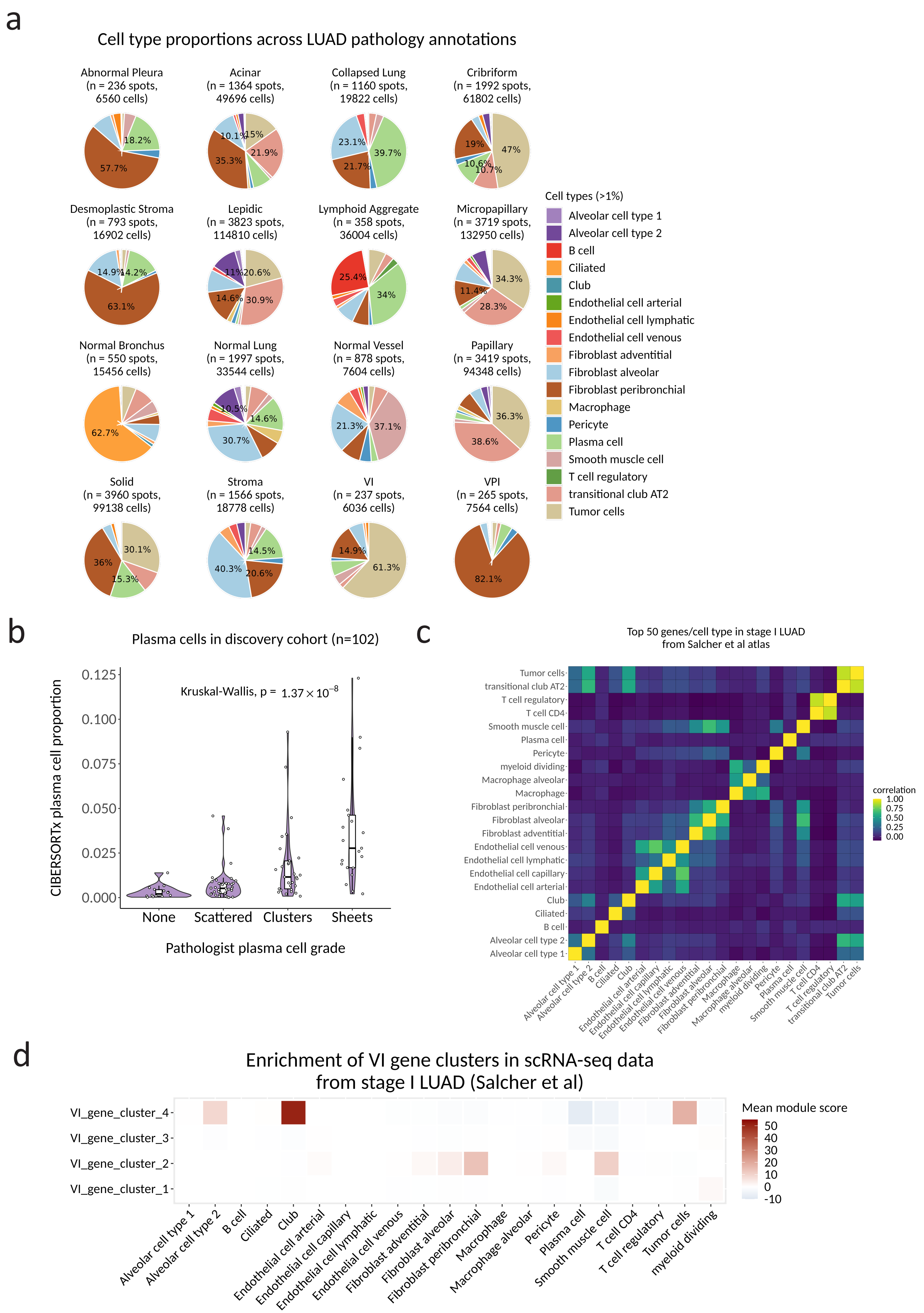

Supplementary Fig. 5. Cell type deconvolution of stRNA-seq and bulk RNA-seq.

Supplementary Fig. 5. Cell type deconvolution of stRNA-seq and bulk RNA-seq. a. Cell type proportions predicted by deconvolution of the stRNA-seq data (n=15 samples) across LUAD pathology annotations. **b**. Proportions of plasma cells estimated by CIBERSORTx deconvolution from bulk RNA-seq in the discovery cohort, stratified by pathologist annotated plasma cell grade (n=102 tumors with annotations). Boxplots display the median, interquartile range (25th–75th percentiles), and whiskers extending to 1.5× the interquartile range. Violin plots depict the distribution of plasma cell proportions for each category, and points represent individual tumors. Statistical significance was assessed using a two-sided Kruskal-Wallis test. **c**. Correlation of cell type marker expression profiles derived from the Salcher lung cancer stRNA-seq atlas. For each annotated cell type in stage I LUAD, the top 50 differentially expressed marker genes were identified, and scaled RNA expression was averaged across all cells of that type. Heatmap values represent Pearson correlation coefficients between mean marker expression profiles for each pair of cell types. Only cell types detected in at least 20% of stRNA-seq samples were included. **d.** Enrichment of VI gene clusters in the Salcher et al stage I LUAD scRNA-seq atlas across the cell types that were used for stRNA-seq deconvolution. Source data are provided as a Source Data file.

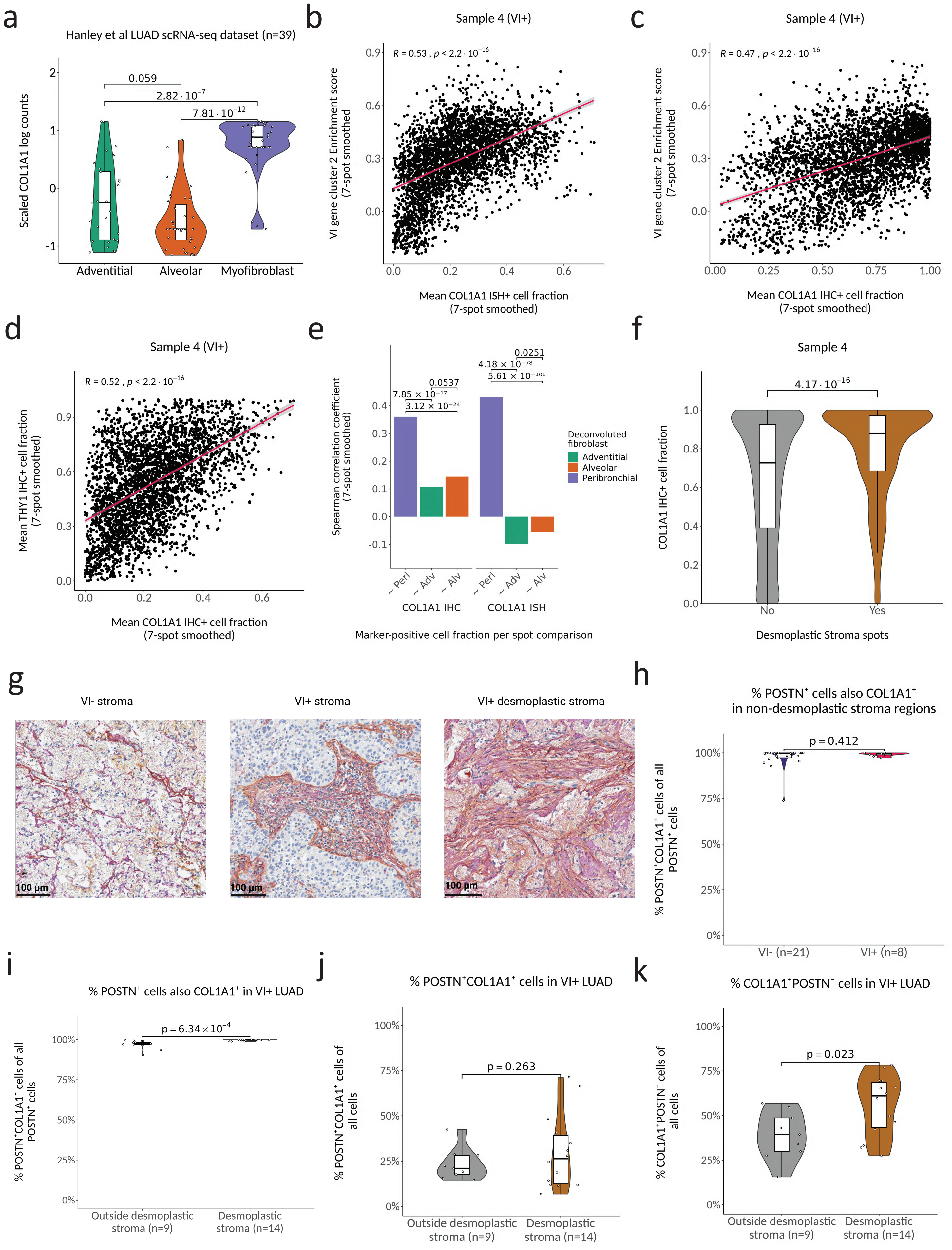

Supplementary Fig. 6. IHC and ISH validation of a key VI cluster 2 gene and association with deconvoluted myofibroblasts and desmoplastic stroma.

Supplementary Fig. 6. IHC and ISH validation of a key VI cluster 2 gene and association with deconvoluted myofibroblasts and desmoplastic stroma. Boxplots display the median, interquartile range (25th–75th percentiles), and whiskers extending to 1.5× the interquartile range. Violin plots depict value distributions, and points represent individual samples or spatial spots unless otherwise noted. **a.** *COL1A1* gene expression in Hanley et al. 2023 fibroblast subpopulations (n=39 samples). For each sample, *COL1A1* log counts were averaged across single cells within each fibroblast subpopulation and z-score normalized within sample. P values were calculated using two-sided Wilcoxon rank-sum tests. **b-c.** Correlation between the VI cluster 2 gene expression per stRNA-seq spot in sample 4 and (b) ISH RNAscope COL1A1^{+} cell fraction or (c) COL1A1^{+} cell fraction by IHC on adjacent sections. The per spot values are spatially smoothed using the observed values of the six most closely neighboring spots (7-spot smoothed). The solid lines indicate linear regression fits with the shaded region representing the 95% CIs. Spearman rank correlation coefficient and the associated two-sided P values are shown. **d.** Correlation between the predicted fraction of COL1A1^{+} cells in stRNA-seq sample 4 and fraction of THY-1^{+} cells by IHC per spot on an adjacent section. The solid line indicates the linear regression fit, with the shaded region representing the 95% CI. Spearman rank correlation coefficient and the associated two-sided P value are shown. **e.** Correlation between predicted myofibroblast fraction in sample 4 (with 7-spot smoothing) and COL1A1^{+} cell fractions measured by either IHC or ISH (also 7-spot smoothed). Bars indicate Spearman's rank correlation coefficient computed on 7-spot spatially smoothed values. All individual correlations were significant by two-sided Spearman rank testing. P values shown compare correlation coefficients across fibroblast subpopulations and were calculated using Steiger's Z test for dependent correlations. **f.** Association of COL1A1^{+} cell fraction by IHC with pathologist annotated desmoplastic stroma spots. P value was calculated using a two-sided Wilcoxon rank-sum test. **g.** Example COL1A1/POSTN dual IHC images of a non-desmoplastic stroma region from VI- LUAD (left), non-desmoplastic stroma region from VI^{+} LUAD (middle), and desmoplastic stroma region from VI^{+} LUAD (right). COL1A1 is labeled with a purple chromogen, POSTN with a yellow chromogen, and direct co-localization appears red. **h-k.** Quantification of fibroblast populations by IHC in pathologist-annotated regions. Panels show (h) fraction of POSTN^{+}COL1A1^{+} myofibroblasts measured by IHC out of all POSTN^{+} cells in pathologist annotated non-desmoplastic stroma regions stratified by VI status (n=29 regions, 21

VI^-, 8 VI^+), (i) fraction of $POSTN^+COL1A1^+$ myofibroblasts measured by IHC out of all $POSTN^+$ cells in desmoplastic stroma regions (n=14 regions) vs. outside desmoplastic stroma regions (n=9 regions) in VI^+ LUAD (n=9 tumors), (j) fraction of $POSTN^+COL1A1^+$ myofibroblasts measured by IHC out of all cells in desmoplastic stroma regions (n=14 regions) vs. outside desmoplastic stroma regions (n=9 regions) in VI^+ LUAD (n=9 tumors), and (k) fraction of $COL1A1^+POSTN^-$ cells measured by IHC out of all cells in desmoplastic stroma regions (n=14 regions) vs. outside desmoplastic stroma regions (n=9 regions) in VI^+ LUAD (n=9 tumors). Statistical significance was assessed using linear mixed-effects models with cell fraction as the dependent variable, VI status (panel (h)) or region (panel (i-k)) as a fixed effect, and tumor sample as a random effect. Two-sided Wald t tests were used, and no adjustment for multiple comparisons was applied. Source data are provided as a Source Data file.

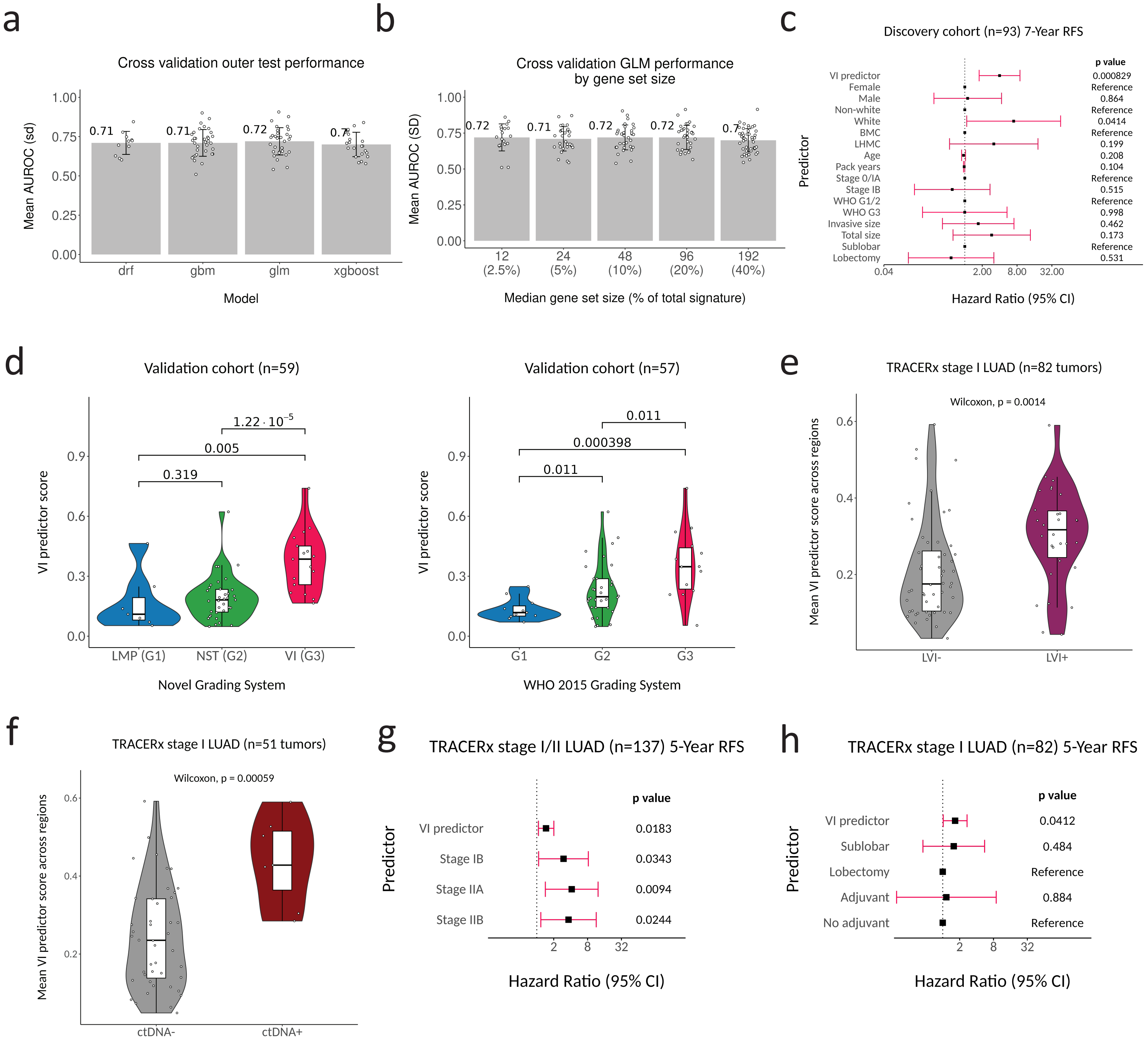

Supplementary Figure 7. VI predictor development and validation.

Supplementary Fig. 7. VI predictor development and validation. Boxplots display the median, interquartile range (25th–75th percentiles), and whiskers extending to 1.5× the interquartile range. Violin plots depict value distributions, and points represent individual tumors unless otherwise noted. **a**. Cross-validation performance of four AutoML model classes evaluated on held-out outer test folds. Shown are mean AUROCs across all outer cross-validation folds (n=100) for distributed random forest (drf), gradient boosting machine (gbm), generalized linear model (glm), and extreme gradient boosting (xgboost). Error bars represent ± one standard deviation (SD) of AUROC across folds. **b.** Cross-validation performance of the glm model by median gene set size (as a percentage of the total VI signature). Error bars represent ± one standard deviation (SD) of AUROC across all cross-validation folds (n=100). **c.** Association of VI predictor with 7-year RFS in the discovery cohort after adjustment for clinical covariates. HRs and 95% CIs were estimated using a multivariable Cox proportional hazards regression model (n=93 tumors, n=15 events). The HR for the VI predictor was estimated per standard deviation increase. Covariates included gender, race, collection site, age, smoking pack years, TNM stage (8th edition), WHO grade, invasive and total tumor size, and surgical procedure. Patients with at least one missing value (n=10) for race (n=2), pack years (n=7), or those with mucinous tumors (n=3) were excluded. Some patients had overlap in missing variables. P values are two-sided Wald test P values derived from the Cox model. Given the low number of recurrences relative to the number of covariates, this analysis is intended as confirmatory rather than as a fully powered prognostic model. **d**. VI predictor association with novel (left) and World Health Organization (WHO) 2015 (right) grading systems in the validation cohort (n=60 tumors). For the WHO 2015 analysis, mucinous tumors (n=2) were removed from grade 3 as this grading system excludes mucinous tumors. **e**. Association of VI predictor scores with LVI^{+} tumors and **f**. patients' preoperative ctDNA status in stage I LUAD RNA-seq data from the TRACERx cohort (n=82 tumors). For each tumor, the VI predictor was averaged across available regions. P values were calculated using two-sided Wilcoxon rank-sum tests. **g.** Association of VI predictor with TRACERx 5-year RFS in stage I and stage II LUAD (n=137 tumors, n=38 events). **h.** Association of VI predictor with TRACERx 5-year RFS in stage I LUAD (n=82 tumors, n=17 events). For both analyses, HRs and 95% CIs were estimated using a multivariable Cox proportional hazards regression model with the HR for the VI predictor estimated per standard deviation increase. TNM stage (7th edition) was included as a covariate in

(g) and surgical procedure and receipt of adjuvant therapy were included as covariates in (h). P values are two-sided Wald test P values. Source data are provided as a Source Data file.

Supplementary Tables

Table S1. Clinical characteristics of resected stage I LUAD tumors from the discovery cohort (n=192 tumors) using the novel histopathology classification.

	LMP	NST	VI	p value
**n**	39	96	57	
**TNM stage 8th edition (%)**				$8.72x10^{-11}$
0	3 (7.7)	0 (0.0)	0 (0.0)	
IA1	27 (69.2)	33 (34.4)	6 (10.5)	
IA2	9 (23.1)	36 (37.5)	18 (31.6)	
IA3	0 (0.0)	9 (9.4)	7 (12.3)	
IB	0 (0.0)	18 (18.8)	26 (45.6)	
**Age (mean (SD))**	68.56 (9.72)	66.55 (9.68)	67.64 (9.41)	0.514
**Gender (%)**				0.037
Female	28 (71.8)	69 (71.9)	30 (52.6)	
Male	11 (28.2)	27 (28.1)	27 (47.4)	
**Race (%)**				0.217
Asian	3 (7.7)	3 (3.1)	1 (1.8)	
Black / African American	3 (7.7)	14 (14.6)	13 (22.8)	
Hispanic / Latino	2 (5.1)	0 (0.0)	2 (3.5)	
Unknown	1 (2.6)	2 (2.1)	1 (1.8)	
White	30 (76.9)	77 (80.2)	40 (70.2)	
**Pack years (mean (SD))**	37.94 (37.84)	45.06 (37.97)	39.10 (22.26)	0.435
**Smoking status (%)**				0.093
Current	8 (20.5)	33 (34.4)	25 (43.9)	
Former	22 (56.4)	50 (52.1)	28 (49.1)	
Never	9 (23.1)	11 (11.5)	4 (7.0)	
Unknown	0 (0.0)	2 (2.1)	0 (0.0)	
**Total size (cm) (mean (SD))**	1.47 (0.54)	1.78 (0.77)	2.12 (0.72)	$1.02x10^{-4}$
**Invasive size (cm) (mean (SD))**	0.68 (0.50)	1.35 (0.68)	1.75 (0.67)	$8.25x10^{-13}$
**% Lepidic (mean (SD))**	62.82 (24.89)	24.48 (27.09)	15.26 (21.10)	$6.06x10^{-17}$
**% Acinar (mean (SD))**	24.87 (22.17)	31.56 (28.08)	32.63 (29.67)	0.349
**% Papillary (mean (SD))**	11.92 (22.05)	13.12 (21.32)	7.19 (14.30)	0.191
**% Solid (mean (SD))**	0.00 (0.00)	16.35 (31.54)	23.95 (31.93)	$3.16x10^{-4}$
**% Micropapillary (mean (SD))**	0.00 (0.00)	5.73 (12.38)	9.56 (17.56)	0.002
**% Cribriform (mean (SD))**	0.38 (1.35)	8.65 (18.67)	9.91 (18.46)	0.014
**LI (%)**	0 (0.0)	24 (25.0)	25 (43.9)	$8.04x10^{-6}$
**VPI (%)**	0 (0.0)	17 (17.7)	24 (42.1)	$2.30x10^{-6}$
**STAS (%)**	0 (0.0)	48 (50.0)	33 (57.9)	$1.11x10^{-8}$
**Institution (%)**				0.583

LHMC	10 (25.6)	28 (29.2)	20 (35.1)	
BMC	29 (74.4)	68 (70.8)	37 (64.9)	
**Procedure (%)**				0.379
Lobe	18 (46.2)	58 (60.4)	38 (66.7)	
Segment	4 (10.3)	7 (7.3)	3 (5.3)	
Wedge	17 (43.6)	31 (32.3)	16 (28.1)	

Note: Continuous variables are summarized as mean ± standard deviation and were compared across groups using two-sided one-way analysis of variance (ANOVA). Categorical variables are summarized as counts (percentages) and were compared using two-sided Pearson's χ^2 test. P values were calculated independently for each variable and were not adjusted for multiple comparisons. Abbreviations: LMP, low malignant potential; NST, no special type; VI, vascular invasion; LI, lymphatic invasion; VPI, visceral pleural invasion; STAS, spread through air spaces; LHMC, Lahey Hospital and Medical Center; BMC, Boston Medical Center. Source data are provided as a Source Data file.

Table S2. Clinical characteristics of resected stage I LUAD tumors with RNA-seq (post-QC) from the discovery cohort (n=103 tumors) using the novel histopathology classification.

	LMP	NST	VI	p value
**n**	23	55	25	
**TNM stage 8th edition (%)**				7.01×10^{-7}
0	3 (13.0)	0 (0.0)	0 (0.0)	
IA1	15 (65.2)	21 (38.2)	1 (4.0)	
IA2	5 (21.7)	17 (30.9)	9 (36.0)	
IA3	0 (0.0)	6 (10.9)	1 (4.0)	
IB	0 (0.0)	11 (20.0)	14 (56.0)	
**Age (mean (SD))**	67.76 (11.39)	65.73 (10.11)	66.23 (7.92)	0.715
**Gender (%)**				0.099
Female	15 (65.2)	40 (72.7)	12 (48.0)	
Male	8 (34.8)	15 (27.3)	13 (52.0)	
**Race (%)**				0.177
Asian	2 (8.7)	2 (3.6)	1 (4.0)	
Black / African American	2 (8.7)	11 (20.0)	7 (28.0)	
Hispanic / Latino	2 (8.7)	0 (0.0)	0 (0.0)	
Unknown	0 (0.0)	1 (1.8)	1 (4.0)	
White	17 (73.9)	41 (74.5)	16 (64.0)	
**Pack years (mean (SD))**	35.50 (36.81)	43.77 (34.45)	36.37 (20.05)	0.494
**Smoking status (%)**				0.221
Current	4 (17.4)	18 (32.7)	12 (48.0)	
Former	13 (56.5)	30 (54.5)	11 (44.0)	
Never	6 (26.1)	6 (10.9)	2 (8.0)	
Unknown	0 (0.0)	1 (1.8)	0 (0.0)	
**Total size (cm) (mean (SD))**	1.34 (0.43)	1.75 (0.75)	2.00 (0.70)	0.005
**Invasive size (cm) (mean (SD))**	0.63 (0.46)	1.28 (0.60)	1.63 (0.57)	9.10×10^{-8}
**% Lepidic (mean (SD))**	63.48 (25.91)	25.27 (27.48)	16.60 (20.55)	3.63×10^{-9}
**% Acinar (mean (SD))**	23.48 (22.69)	26.45 (23.47)	36.00 (28.72)	0.169
**% Papillary (mean (SD))**	12.83 (24.90)	16.09 (23.23)	8.40 (9.76)	0.323
**% Solid (mean (SD))**	0.00 (0.00)	15.45 (30.60)	21.60 (32.04)	0.021
**% Micropapillary (mean (SD))**	0.00 (0.00)	6.00 (13.69)	8.60 (17.11)	0.068
**% Cribriform (mean (SD))**	0.22 (1.04)	10.55 (20.22)	8.80 (13.01)	0.038
**LI (%)**	0 (0.0)	14 (25.5)	13 (52.0)	2.27×10^{-4}
**VPI (%)**	0 (0.0)	11 (20.0)	13 (52.0)	8.09×10^{-5}
**STAS (%)**	0 (0.0)	28 (50.9)	12 (48.0)	8.02×10^{-5}
**Institution (%)**				0.093

LHMC	6 (26.1)	5 (9.1)	6 (24.0)	
BMC	17 (73.9)	50 (90.9)	19 (76.0)	
**Procedure (%)**				0.23
Lobe	9 (39.1)	35 (63.6)	12 (48.0)	
Segment	2 (8.7)	1 (1.8)	2 (8.0)	
Wedge	12 (52.2)	19 (34.5)	11 (44.0)	

Note: Continuous variables are summarized as mean ± standard deviation and were compared across groups using two-sided one-way analysis of variance (ANOVA). Categorical variables are summarized as counts (percentages) and were compared using two-sided Pearson's χ^2 test. P values were calculated independently for each variable and were not adjusted for multiple comparisons. The data are shown as the number and (%) unless otherwise indicated. Abbreviations: LMP, low malignant potential; NST, no special type; VI, vascular invasion; LI, lymphatic invasion; VPI, visceral pleural invasion; STAS, spread through air spaces; LHMC, Lahey Hospital and Medical Center; BMC, Boston Medical Center. Source data are provided as a Source Data file.

Table S3. Clinical characteristics of resected early-stage LUAD tumors with stRNA-seq (post-QC) from the validation cohort (n=15 samples).

Sample ID	Batch	8th Stage	Novel Grade	Age	Gender	Smoking Status	RNA-seq	VI (Capture Frame)
1A	1	IB	VI	60-69	M	Former	Yes	1
1B	2	IB	VI	60-69	M	Former	Yes	0
2B	2	IA3	VI	60-69	F	Current	Yes	0
2A	2	IA3	VI	60-69	F	Current	Yes	0
3	2	IA1	NST	70-79	M	Former	Yes	0
4	2	IB	VI	60-69	M	Former	Yes	1
5	2	IA1	NST	70-79	M	Former	Yes	0
7	2	IA1	LMP	60-69	F	Current	Yes	0
8	1	IB	VI	50-59	M	Former	Yes	0
9	2	IA2	VI	60-69	F	Current	Yes	1
10	2	IA2	NST	60-69	M	Former	Yes	0
11	1	IIA	NST	60-69	F	Former	Yes	0
12	2	IB	NST	60-69	F	Former	Yes	0
13	1	IA1	NST	60-69	F	Former	Yes	0
14	2	IA2	NST	70-79	F	Former	Yes	0

Note: Abbreviations: LMP, low malignant potential; NST, no special type; VI, vascular invasion.
Source data are provided as a Source Data file.

Table S4. Clinical characteristics of resected stage I LUAD tumors with IHC (n=20 tumors) using the novel histopathology classification.

	LMP	NST	VI	p value
**n**	6	5	9	
**TNM stage 8th edition (%)**				0.122
IA2	0 (0.0)	1 (20.0)	4 (44.4)	
IA3	3 (50.0)	2 (40.0)	0 (0.0)	
IB	3 (50.0)	2 (40.0)	5 (55.6)	
**Age (mean (SD))**	72.50 (6.95)	68.00 (11.73)	72.00 (5.39)	0.583
**Gender (%)**				0.393
Female	4 (66.7)	3 (60.0)	3 (33.3)	
Male	2 (33.3)	2 (40.0)	6 (66.7)	
**Race (%)**				0.101
Asian	0 (0.0)	2 (40.0)	0 (0.0)	
Other	0 (0.0)	0 (0.0)	1 (11.1)	
White	6 (100.0)	3 (60.0)	8 (88.9)	
**Smoker (%)**				0.489
Current	0 (0.0)	0 (0.0)	2 (22.2)	
Former	4 (66.7)	4 (80.0)	6 (66.7)	
Never	2 (33.3)	1 (20.0)	1 (11.1)	
**Procedure (%)**	0 (0.0)	0 (0.0)	1 (11.1)	0.526
Lobe	6 (100.0)	5 (100.0)	1 (89.9)	
Wedge	0 (0.0)	0 (0.0)	1 (11.1)	

Note: Continuous variables are summarized as mean ± standard deviation and were compared across groups using two-sided one-way analysis of variance (ANOVA). Categorical variables are summarized as counts (percentages) and were compared using two-sided Pearson's χ^2 test. P values were calculated independently for each variable and were not adjusted for multiple comparisons. Abbreviations: LMP, low malignant potential; NST, no special type; VI, vascular invasion; all cases were from Inova Schar Cancer Institute. Source data are provided as a Source Data file.

Table S5. Clinical characteristics of resected stage I LUAD tumors with RNA-seq (post-QC) from the validation cohort (n=60 tumors) using the novel histopathology classification.

	LMP	NST	VI	p value
**n**	7	36	17	
**TNM stage 8th edition (%)**				0.016
IA1	6 (85.7)	12 (34.3)	2 (11.8)	
IA2	1 (14.3)	9 (25.7)	3 (17.6)	
IA3	0 (0.0)	6 (17.1)	3 (17.6)	
IB	0 (0.0)	8 (22.9)	9 (52.9)	
**Age (mean (SD))**	65.57 (8.50)	66.31 (7.30)	61.76 (6.44)	0.107
**Gender (%)**				0.205
Female	6 (85.7)	19 (52.8)	8 (47.1)	
Male	1 (14.3)	17 (47.2)	9 (52.9)	
**Pack years (mean (SD))**	31.43 (11.80)	49.53 (25.19)	51.65 (17.91)	0.114
**Smoking status (%)**				0.408
Current	2 (28.6)	13 (36.1)	9 (52.9)	
Former	5 (71.4)	23 (63.9)	8 (47.1)	
**Total size (cm) (mean (SD))**	1.90 (0.76)	1.93 (0.87)	2.22 (0.90)	0.484
**Invasive size (cm) (mean (SD))**	0.81 (0.38)	1.68 (0.95)	2.06 (0.84)	0.01
**% Lepidic (mean (SD))**	55.71 (22.25)	22.78 (29.46)	9.12 (14.71)	6.88×10^{-4}
**% Acinar (mean (SD))**	26.43 (22.12)	29.31 (26.68)	20.29 (24.46)	0.494
**% Papillary (mean (SD))**	17.86 (30.80)	19.44 (28.63)	8.82 (10.97)	0.361
**% Solid (mean (SD))**	0.00 (0.00)	5.00 (11.46)	46.47 (37.03)	5.38×10^{-8}
**% Micropapillary (mean (SD))**	0.00 (0.00)	10.28 (20.91)	4.71 (10.23)	0.267
**% Cribriform (mean (SD))**	0.00 (0.00)	13.33 (27.67)	10.59 (20.30)	0.415
**LI (%)**	0 (0.0)	17 (47.2)	9 (52.9)	0.045
**VPI (%)**	0 (0.0)	5 (13.9)	7 (41.2)	0.025
**STAS (%)**	0 (0.0)	14 (38.9)	10 (58.8)	0.027
**Procedure (%)**				0.79
Lobe	6 (85.7)	27 (79.4)	14 (82.4)	
Segment	1 (14.3)	2 (5.9)	1 (5.9)	
Wedge	0 (0.0)	5 (14.7)	2 (11.8)	

Note: Continuous variables are summarized as mean ± standard deviation and were compared across groups using two-sided one-way analysis of variance (ANOVA). Categorical variables are summarized as counts (percentages) and were compared using two-sided Pearson's χ^2 test. P values were calculated independently for each variable and were not adjusted for multiple comparisons. Abbreviations: LMP, low malignant potential; NST, no special type; VI, vascular invasion; LI, lymphatic invasion; VPI, visceral pleural invasion; STAS, spread through air spaces; all cases were from Lahey Hospital and Medical Center. Source data are provided as a Source Data file.

Table S6. Clinical characteristics of pre-surgical biopsies from stage I LUAD tumors (n=24 biopsies, 12 matched resections) using the novel histopathology classification.

	LMP	NST	VI	p value
**n**	6	6	12	
**TNM stage 8th edition (%)**				0.199
IA2	1 (16.7)	3 (50.0)	4 (33.3)	
IA3	4 (66.7)	2 (33.3)	2 (16.7)	
IAB	1 (16.7)	1 (16.7)	6 (60.0)	
**Age (mean (SD))**	70.17 (6.88)	70.17 (11.29)	69.25 (8.28)	0.968
**Gender (%)**				0.777
Female	4 (66.7)	3 (50.0)	6 (50.0)	
Male	2 (33.3)	3 (50.0)	6 (50.0)	
**Race (%)**				0.271
Asian	0 (0.0)	1 (16.7)	0 (0.0)	
Black	0 (0.0)	0 (0.0)	1 (8.3)	
Other	0 (0.0)	0 (0.0)	3 (25.0)	
White	6 (100.0)	5 (83.3)	8 (66.7)	
**Smoking status (%)**				0.604
Current	0 (0.0)	0 (0.0)	2 (16.7)	
Former	5 (83.3)	5 (83.3)	7 (58.3)	
Never	1 (16.7)	1 (16.7)	3 (25.0)	
**Procedure (%)**				0.407
Lobe	4 (66.7)	5 (83.3)	11 (91.7)	
Wedge	2 (33.3)	1 (16.7)	1 (8.3)	
**Biopsy type (%)**				0.130
Fine needle aspiration	0 (0.0)	0 (0.0)	1 (8.3)	
Forceps	0 (0.0)	0 (0.0)	3 (25.0)	
Unknown	0 (0.0)	0 (0.0)	3 (25.0)	
Needle	6 (100.0)	6 (100.0)	5 (41.7)	

Note: Continuous variables are summarized as mean ± standard deviation and were compared across groups using two-sided one-way analysis of variance (ANOVA). Categorical variables are summarized as counts (percentages) and were compared using two-sided Pearson's χ^2 test. P values were calculated independently for each variable and were not adjusted for multiple comparisons. Abbreviations: LMP, low malignant potential; NST, no special type; VI, vascular invasion; all cases were from Inova Schar Cancer Institute. Source data are provided as a Source Data file.
