# Supplementary material for: Vascular invasion-associated gene expression is detectable in pre-surgical biopsies of stage I lung adenocarcinoma
Source: Nat Commun. 2026 Mar 24;17:2581. doi: 10.1038/s41467-026-70600-2 (PMC13013712; doi:10.1038/s41467-026-70600-2)
Supplement: Supplementary file 3 — Reporting Summary [file 41467_2026_70600_MOESM3_ESM.pdf]

nature portfolio

Corresponding author(s): Marc E. Lenburg, PhD

Last updated by author(s): 5/16/2025

Reporting Summary

Nature Portfolio wishes to improve the reproducibility of the work that we publish. This form provides structure for consistency and transparency in reporting. For further information on Nature Portfolio policies, see our Editorial Policies and the Editorial Policy Checklist.

Statistics

For all statistical analyses, confirm that the following items are present in the figure legend, table legend, main text, or Methods section.

n/a	Confirmed	
☐	☒	The exact sample size (*n*) for each experimental group/condition, given as a discrete number and unit of measurement
☐	☒	A statement on whether measurements were taken from distinct samples or whether the same sample was measured repeatedly
☐	☒	The statistical test(s) used AND whether they are one- or two-sided<br>*Only common tests should be described solely by name; describe more complex techniques in the Methods section.*
☐	☒	A description of all covariates tested
☐	☒	A description of any assumptions or corrections, such as tests of normality and adjustment for multiple comparisons
☐	☒	A full description of the statistical parameters including central tendency (e.g. means) or other basic estimates (e.g. regression coefficient) AND variation (e.g. standard deviation) or associated estimates of uncertainty (e.g. confidence intervals)
☐	☒	For null hypothesis testing, the test statistic (e.g. *F*, *t*, *r*) with confidence intervals, effect sizes, degrees of freedom and *P* value noted<br>*Give P values as exact values whenever suitable.*
☒	☐	For Bayesian analysis, information on the choice of priors and Markov chain Monte Carlo settings
☐	☒	For hierarchical and complex designs, identification of the appropriate level for tests and full reporting of outcomes
☐	☒	Estimates of effect sizes (e.g. Cohen's *d*, Pearson's *r*), indicating how they were calculated

Our web collection on statistics for biologists contains articles on many of the points above.

Software and code

Policy information about availability of computer code

Data collection	FASTQ files for RNA-Seq experiments were demultiplexed using BaseSpace (Illumina).<br>RNA-seq processing pipeline software (https://github.com/compbiomed/RNA_Seq/blob/master/RNA_Seq.nf): nextflow v21.10.16, star v2.6.0c, rsem v1.3.1, FastQC v0.11.7, Picard tools v2.18.11, GATK v3.8-1, rseqc v3.0.0, multiqc v1.6, samtools v1.9<br>Spatial transcriptomics demultiplexing and processing pipeline software (https://www.10xgenomics.com/support/software/space-ranger/downloads/previous-versions): Space Ranger v1.3.1, bcl2fastq v2.20
Data analysis	The analysis scripts to reproduce the findings reported in this study are archived on Zenodo (https://doi.org/10.5281/zenodo.18395867)

For manuscripts utilizing custom algorithms or software that are central to the research but not yet described in published literature, software must be made available to editors and reviewers. We strongly encourage code deposition in a community repository (e.g. GitHub). See the Nature Portfolio guidelines for submitting code & software for further information.

Data

Policy information about availability of data

All manuscripts must include a data availability statement. This statement should provide the following information, where applicable:

- Accession codes, unique identifiers, or web links for publicly available datasets
- A description of any restrictions on data availability
- For clinical datasets or third party data, please ensure that the statement adheres to our policy

All bulk RNA sequencing data and spatial transcriptomics data generated in this study has been deposited in the NCBI Gene Expression Omnibus (GEO) under series GSE273528 [https://www.ncbi.nlm.nih.gov/geo/query/acc.cgi?acc=GSE273528]. Publicly available data used in this study are available as follows: Uppsala RNA-seq and associated clinical data are available under GEO series GSE81089 [https://www.ncbi.nlm.nih.gov/geo/query/acc.cgi?acc=GSE81089]. TCGA RNA-seq and associated clinical data are available on the National Cancer Institute Genomic Data Commons Data Portal under project TCGA-LUAD [https://portal.gdc.cancer.gov/projects/tcga-luad]. TRACERx RNA-seq and associated clinical data are available on Zenodo under records 7683605 [https://zenodo.org/records/7683605] and 7603386 [https://zenodo.org/records/7603386]. The single-cell lung cancer atlas (LuCA) scRNA-seq data is available on Zenodo under record 6411868 [https://zenodo.org/records/6411868]. Source Data are provided with this paper. All other data supporting the findings of this study are available within the article and its supplementary information files.

Research involving human participants, their data, or biological material

Policy information about studies with human participants or human data. See also policy information about sex, gender (identity/presentation), and sexual orientation and race, ethnicity and racism.

Reporting on sex and gender	Self-identified gender was determined by retrospective chart review and cross-referenced with institutional tumor registry data after approval by the Institutional Review Board at each site (BU/BMC IRB H-37859, Lahey Clinic IRB-518308 & Inova IRB U23-06-5093). A distinction between sex and gender was not part of medical records over the period of time this retrospective review was conducted.
Reporting on race, ethnicity, or other socially relevant groupings	Self-identified race was determined by retrospective chart review and cross-referenced with institutional tumor registry data after approval by the Institutional Review Board at each site (BU/BMC IRB H-37859, Lahey Clinic IRB-518308, & Inova IRB U23-06-5093).
Population characteristics	Population characteristics of the discovery, validation, and pre-surgical biopsy cohorts are detailed in Table S1, S3 and S4 respectively. An experienced thoracic pathologist (E.B.) reviewed all pathology cases. Vascular invasion (VI) was defined as luminal invasion of a vein or muscular artery either within or adjacent to the tumor. Tumor proportions of lepidic, acinar, papillary, micropapillary, and solid patterns were assessed in 5% increments with distinction of simple tubular acinar from complex and cribriform acinar patterns. Adenocarcinoma in situ (AIS) was assigned to purely lepidic tumors ≤3 cm whereas minimally invasive adenocarcinoma (MIA) was diagnosed when non-lepidic foci measured ≤0.5 cm as per World Health Organization (WHO) criteria. WHO- 2021 grade was defined as G1, lepidic predominant with <20% high-grade patterns; G2, acinar or papillary predominant with <20% high-grade patterns; and G3, ≥20% high-grade patterns (solid, micropapillary and/or complex glands). Low malignant potential adenocarcinoma (LMP) was assigned as previously described24. LMP tumors were non-mucinous adenocarcinoma measuring ≤3 cm in total size, with ≥15% lepidic growth, and without nonpredominant high-grade patterns (≥10% cribriform, ≥5% micropapillary, ≥5% solid), >1 mitosis per 2 mm2, vascular, lymphatic or visceral pleural invasion, STAS or necrosis. AIS/MIA and LMP were analyzed together due to their identical outcome (100% 10-year disease specific survival). One LMP in the discovery cohort recurred after wedge-resection with a positive surgical margin. The tumor recurred at the staple line and was treated with SBRT, resulting in prolonged survival (over 10 years) without further recurrence or metastasis. No special type (NST) designation was given for all other tumors not classified as VI or LMP. Stage assignments were retrospectively made using the 8th edition of the American Joint Committee on Cancer (AJCC).
Recruitment	Cases were identified by retrospective chart review and cross-referenced with institutional tumor registry data after approval by the Institutional Review Board at each site (BU/BMC IRB H-37859, Lahey Clinic IRB-518308, Inova IRB U23-06-5093).
Ethics oversight	The Boston Medical Center (BMC), Lahey Hospital & Medical Center (LHMC), and Inova Schar Cancer Institute Institutional Review Boards approved this study (BU/BMC IRB H-37859; Lahey Clinic IRB-518308; Inova U23-06-5093).

Note that full information on the approval of the study protocol must also be provided in the manuscript.

Field-specific reporting

Please select the one below that is the best fit for your research. If you are not sure, read the appropriate sections before making your selection.

☒ Life sciences ☐ Behavioural & social sciences ☐ Ecological, evolutionary & environmental sciences

For a reference copy of the document with all sections, see nature.com/documents/nr-reporting-summary-flat.pdf

Life sciences study design

All studies must disclose on these points even when the disclosure is negative.

Sample size	A discovery cohort consisting of 192 resected tumors from 192 patients with 8th edition TNM stage 0/1 LUAD and not treated with neoadjuvant or adjuvant therapy were included in this study. Cases were from Boston Medical Center (BMC) and Lahey Hospital & Medical

Center (LHMC). Age, sex, self-identified race, extent of cigarette smoke exposure, extent of resection, total tumor size, tumor laterality, absence of lymph node metastasis, time to recurrence, death, and cause of death were determined by retrospective chart review and cross-referenced with institutional tumor registry data after approval by the Institutional Review Board at each site (BU/BMC IRB H-37859 & Lahey Clinic IRB-518308). Tumors measuring >4 cm total size were not included as subsets of these were treated with adjuvant therapy within this historic cohort.
A validation cohort consisting of 61 resected tumors from 60 patients with 8th edition TNM stage I/II LUAD and not treated with neoadjuvant or adjuvant therapy were included in this study. All samples were selected to be stage IA/IB at time of collection except for one tumor that was upstaged to stage IIA under the 8th TNM edition.
Cases were from LHMC. Age, sex, self-identified race, extent of cigarette smoke exposure, extent of resection, total tumor size, tumor laterality, absence of lymph node metastasis, time to recurrence, death, and cause of death were determined by retrospective chart review and cross-referenced with institutional tumor registry data after approval by the Institutional Review Board (Lahey Clinic IRB-518308). Tumors measuring >4 cm total size were not included as subsets of these were treated with adjuvant therapy within this historic cohort.
RNA-seq was performed on an independent validation cohort consisting of 24 pre-surgical biopsies and 12 matched resected tumors from 24 patients with 8th edition TNM stage I LUAD. All samples were from patients not treated with neoadjuvant or adjuvant therapy and were receiving care at Inova Schar Cancer Institute. Biopsies were collected either via CT-guided needle biopsy, endobronchial ultrasound (EBUS), or navigational bronchoscopy. The determination of novel grade was based upon a representative FFPE block of the resected tumor.

Data exclusions	The discovery cohort tumors (n=192) represented a wide range of sample ages, so RNAseq was first performed on a small pilot batch of 12 samples of varying ages to determine which tumors might yield usable RNAseq data (4/5 of the samples removed post-QC belonged to this pilot batch) by correlating sample characteristics with RNAseq QC measures. Using these data, we selected an additional 96 samples for RNAseq from the discovery cohort that yielded at least 83 ng of library. In total, RNA-seq data was available from 108 tumors (these 96 and the initial 12 tumors profiled in the pilot), with 103 passing quality control (QC described below).<br>For the validation cohort, RNA-seq was performed on 61 tumors, with 60 passing quality control. StRNA-seq was performed on a subset of these 61 tumors and included 16 samples taken from 14 tumors (13 stage I tumors and 1 upstaged stage II tumor). Relevant clinical information on all stRNA-seq samples included can be found in Table S2. Only 8th edition TNM stage I LUAD from the validation cohort was included in the bulk RNA-seq (n=59) analysis.<br>For RNA-seq data, quality metrics were calculated with STAR and RSeQC. EdgeR was used to compute normalized data (library sizes normalized using trimmed mean of M-values). Genes with count per million (cpm) > 1 in at least 10% of samples were retained for further analysis. Samples were excluded from the discovery and validation cohorts if the transcript integrity number (TIN) calculated by RSeQC was > 2 standard deviations from the mean TIN.<br>For spatial transcriptomics data, all samples were selected to be stage IA/IB at the time of collection except for one that was upstaged to stage IIA under the 8th TNM edition (this sample was excluded from the bulk RNA-seq validation cohort when evaluating the performance of VI predictor scores). Samples were chosen based on 1) the presence of pathological features of interest (e.g., VI foci, representative LUAD histologic patterns) and 2) more than 50% of RNA fragments being greater than 200 nucleotides (DV200) after extraction with the AllPrep DNA/RNA Universal Kit (Qiagen) during bulk RNA-seq library preparation. Samples were removed (n=1) if tissue loss occured during the workflow. Low-quality spots and spots not covered by tissue were filtered if < 250 genes were detected per spot.
Replication	The results presented were derived in a discovery cohort of resected tumor samples and replicated in validation cohort of resected tumor samples and another pre-surgical biopsy validation cohort.
Randomization	Randomization was not relevant to our retrospective study, as the goal was to discover biomarkers to predict angioinvasive lung adenocarcinoma. Future prospective studies, comparing outcomes related to specific treatment based on biomarker prediction will require randomization.
Blinding	Blinding was not relevant to our retrospective study, as the goal was to discover biomarkers to predict angioinvasive lung adenocarcinoma. Future prospective studies, where the biomarkers are rigorously tested, will require blinding.

Reporting for specific materials, systems and methods

We require information from authors about some types of materials, experimental systems and methods used in many studies. Here, indicate whether each material, system or method listed is relevant to your study. If you are not sure if a list item applies to your research, read the appropriate section before selecting a response.

Materials & experimental systems

n/a	Involved in the study	
☒	☐	Antibodies
☒	☐	Eukaryotic cell lines
☒	☐	Palaeontology and archaeology
☒	☐	Animals and other organisms
☐	☒	Clinical data
☒	☐	Dual use research of concern
☒	☐	Plants

Methods

n/a	Involved in the study	
☒	☐	ChIP-seq
☒	☐	Flow cytometry
☒	☐	MRI-based neuroimaging

Clinical data

Policy information about clinical studies

All manuscripts should comply with the ICMJE guidelines for publication of clinical research and a completed CONSORT checklist must be included with all submissions.

Clinical trial registration	N/A
Study protocol	Not available, as this was a retrospective study.
Data collection	Cases were reviewed from Boston Medical Center (BMC), Lahey Hospital & Medical Center (LHMC), and Inova Schar Cancer Institute after Institutional Review Board approval at each site (BU/BMC IRB H-37859, Lahey Clinic IRB-518308 & Inova IRB U23-06-5093) in which patient consent was waived as this retrospective study posed no more than minimal risk of harm to subjects and involved no procedures for which written consent is normally required.
Outcomes	Survival assessment was measured as: recurrence free survival (RFS), defined as time from initial surgery to recurrence of resected tumor or time of last follow-up; disease specific survival (DSS), defined as time from surgery to death from recurrence of resected tumor or time of last follow-up; and overall survival (OS), defined as time from surgery to death from any cause or time of last follow-up.

Plants

Seed stocks	*Report on the source of all seed stocks or other plant material used. If applicable, state the seed stock centre and catalogue number. If plant specimens were collected from the field, describe the collection location, date and sampling procedures.*
Novel plant genotypes	*Describe the methods by which all novel plant genotypes were produced. This includes those generated by transgenic approaches, gene editing, chemical/radiation-based mutagenesis and hybridization. For transgenic lines, describe the transformation method, the number of independent lines analyzed and the generation upon which experiments were performed. For gene-edited lines, describe the editor used, the endogenous sequence targeted for editing, the targeting guide RNA sequence (if applicable) and how the editor was applied.*
Authentication	*Describe any authentication procedures for each seed stock used or novel genotype generated. Describe any experiments used to assess the effect of a mutation and, where applicable, how potential secondary effects (e.g. second site T-DNA insertions, mosiacism, off-target gene editing) were examined.*
